# *o2geosocial*: Reconstructing who-infected-whom from routinely collected surveillance data

**DOI:** 10.12688/f1000research.28073.1

**Published:** 2021-01-18

**Authors:** Alexis Robert, Sebastian Funk, Adam J Kucharski

**Affiliations:** 1 Centre for the Mathematical Modelling of Infectious Diseases, London School of Hygiene & Tropical Medicine, London, WC1E 7HT, UK; 2 Department of Infectious Disease Epidemiology, London School of Hygiene & Tropical Medicine, London, WC1E 7HT, UK

**Keywords:** Transmission tree reconstruction, Bayesian statistics, Monte Carlo Markov Chains, outbreaks, R

## Abstract

Reconstructing the history of individual transmission events between cases is key to understanding what factors facilitate the spread of an infectious disease. Since conducting extended contact-tracing investigations can be logistically challenging and costly, statistical inference methods have been developed to reconstruct transmission trees from onset dates and genetic sequences. However, these methods are not as effective if the mutation rate of the virus is very slow, or if sequencing data is sparse.

We developed the package *o2geosocial* to combine variables from routinely collected surveillance data with a simple transmission process model. The model reconstructs transmission trees when full genetic sequences are not available, or uninformative. Our model incorporates the reported age-group, onset date, location and genotype of infected cases to infer probabilistic transmission trees. The package also includes functions to summarise and visualise the inferred cluster size distribution.

The results generated by *o2geosocial* can highlight regions where importations repeatedly caused large outbreaks, which may indicate a higher regional susceptibility to infections. It can also be used to generate the individual number of secondary transmissions, and show the features associated with individuals involved in high transmission events.

The package is available for download from the Comprehensive R Archive Network (CRAN) and GitHub.

## Introduction

The identification of transmission trees and transmission events during infectious disease outbreaks can lead to identifying factors associated with subsequent transmissions ^1– 3^, describing the populations or the areas more vulnerable to importations and transmission ^4– 7^, and quantifying the impact of control measures ^8, 9^. The most straightforward approach to reconstruct who-infected-whom is to carry out patient interviews and establish the previous contacts to connect the reported cases. However, contact-tracing investigations are costly and can be challenging to implement. Statistical methods have therefore been developed to infer transmission trees from routinely collected epidemiological data ^9– 14^.

The Wallinga-Teunis method was first developed to infer probabilistic transmission trees from onset dates and serial intervals in a maximum likelihood framework ^9^. Genetic sequencing of pathogens have since become more common, and new tools such as the R package *outbreaker2* were created to combine the timing of infection and the genetic sequences in order to improve the accuracy of inferred transmission trees ^10, 11, 15– 17^. Nevertheless, the accuracy of these reconstruction methods relies on the proportion of sequenced cases, the quality of the sequences, and the characteristics of the pathogen ^18^. For instance, the measles virus evolves very slowly, and sequences from unrelated cases can be very similar, which makes these methods ineffective for measles outbreaks ^19, 20^.

The package *o2geosocial* was designed to study outbreaks where sequences are uninformative, either because too few cases were sequenced or because the virus evolves too slowly. Building upon the framework presented in *outbreaker2*, *o2geosocial* was developed to infer who-infected-whom from variables routinely collected by surveillance systems, such as the onset date, age, location, and genotype of the cases ^4^. Cases from different genotypes cannot be part of a similar transmission chain since differences in genotype illustrate major variations in their genetic sequences, ^21^. Using age-stratified contact matrices and mobility models, we combined the different variables into a likelihood of connection between cases. In this paper, we first describe the structure of the package. From a use case based on simulated data, we then show how to run the model, evaluate the output, visualise the results of the inference, and customise the input functions to implement different mobility models.

## Methods

### Operation

*o2geosocial* is implemented as an open-source R (version ≥ 3.5.0) package and can be run on all platforms (Windows, Mac, Linux). It incorporates C++ functions into a R framework using Rcpp ^22^. Package dependencies and system requirements are documented in the o2geosocial CRAN repository. A stable version was released on Windows, Mac and Linux operating systems via a CRAN repository. The source code is available through Zenodo ^23^ and the latest development version is available through a Github repository.


# install from CRAN
install.packages("o2geosocial")
# install from Github
install.packages("devtools")
devtools::install_github("alxsrobert/o2geosocial")


The main function of the package, called outbreaker(), uses Monte Carlo Markov Chains (MCMC) to sample from the posterior distribution of the underlying model ^24^. For each case, it infers the infection date, the infector, and the number of missing generations between the case and their infector. It takes five lists as inputs: i) ‘moves’, ii) ‘likelihoods’, iii) ‘priors’, iv) ‘data’, and v) ‘config’. These five lists can be generated and modified using the functions custom_moves(), custom_likelihoods(), custom_priors(), create_config() and outbreaker_data().

### Implementation

The general implementation of *o2geosocial* follows the structure of *outbreaker2* and builds upon it by adding the effect of the location and the age-stratified contact data to the reconstruction of transmission clusters. However, unlike *outbreaker2*, *o2geosocial* does not take genetic sequences as input. It uses genetic groups ( *e.g.* genotype) to exclude connections between cases, *i.e.* two cases cannot be from the same cluster if they belong to different genetic groups ^25^. Therefore, *o2geosocial* is adapted to reconstructing transmission clusters from large datasets where genetic sequences are not informative, either because the mutation rate of the virus is slow, or because sequencing is scarce.

**Figure 1.**
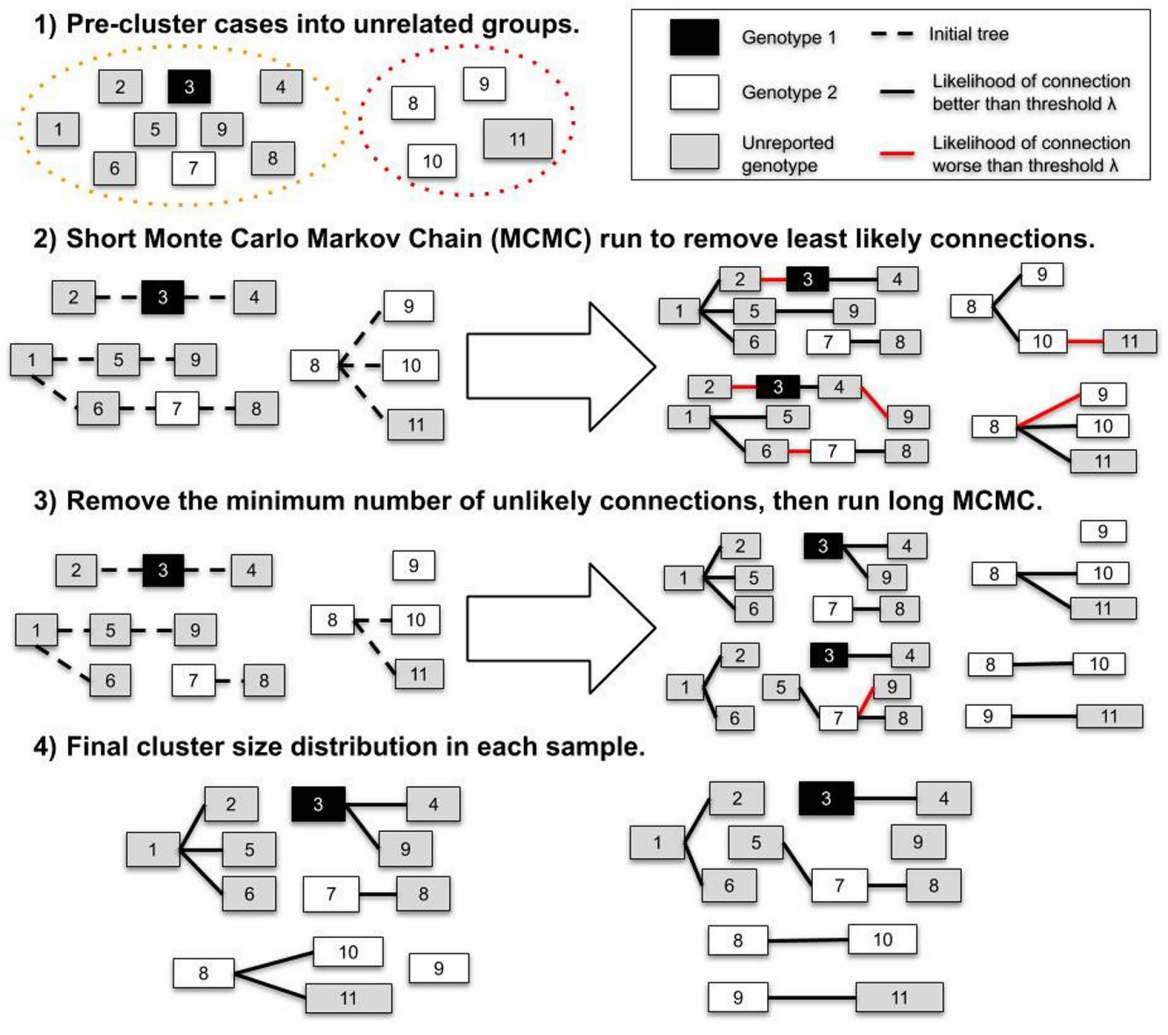
Illustration of the process to estimate the cluster size distribution and the import status of 11 cases. In the first step, cases are split in two groups that do not have overlapping potential infectors (i.e. they were reported in different places, or different times). In step 2, we estimate the minimum number of unlikely transmissions (n) in the samples (right panel). In step 3, we remove n transmissions from the initial tree, and generate samples. Finally, we remove the unlikely connections in each sample of Step 3 and compute the inferred cluster size distribution.

In *o2geosocial*, the number of independent clusters in the dataset is inferred using two different processes ( Figure 1). Firstly, the pre-clustering step aims to group cases before the MCMC runs following three criteria: Only cases reported in a radius of γ km, less than δ days before case i, and from similar or unreported genotype can be classified as potential infectors of i. Cases with overlapping potential infectors, and their potential infectors, are grouped together, and cases from different groups cannot be linked during the MCMC runs. The parameters γ and δ are defined as inputs of the function create_config(). Since surveillance datasets can include cases from unrelated outbreaks, the pre-clustering function was developed to remove impossible connections and speed up the MCMC runs.

Secondly, as cases classified in the same group after the pre-clustering step may come from different clusters, we defined a likelihood threshold *λ* to spot and discard unlikely connections after the MCMC runs: if the likelihood of connection between cases from *j* to *i* is lower than *λ*, the connection is discarded and *i* is an import unrelated to *j*. In *o2geosocial*, the variable *λ* can either be an absolute (the log-likelihood threshold will be *log(λ)*) or a relative value (a quantile of the likelihood of all connections in all samples), and is defined by the variables ‘outlier_threshold’ and ‘outlier_relative’ in create_config().

Finally, unlikely connections between cases can alter the inferred infection dates of cases and bias the transmission trees sampled form the MCMC runs. Therefore, we first run a short MCMC to remove these unlikely connections. From this run we compute *n*, the minimum number of connections with a likelihood lower than *λ* per sampled tree. We then add *n* imports to the starting tree and run a longer MCMC. Lastly, we remove the connections with likelihood lower than *λ* in the final samples and return the infector, infection date and probability of being an import for each case ( Figure 1).

### Likelihood and priors

The functions custom_likelihoods() and custom_priors() can be used to edit each component of the likelihood and priors. By default, there are five components in the likelihood:

***Genotype component***: There can be a maximum of one genotype reported per transmission tree. The genotype of a tree *τ* is the genotype reported for at least one of the cases belonging to *τ*. For each genotyped case *i _gen_* and at every iteration, only cases from trees with the same genotype as *i _gen_*, or without reported genotype can be listed as potential infectors.

Therefore, the genetic component of the likelihood that a case *i* of genotype *g _i_* was infected by a case *j* belonging to the tree *τ* *_j_* is defined as a binary value:

$$G(g_{i},g_{\tau_{j}})\;=\;\left\{ \begin{array}{l} 1\;\text{if}\;g_{i}\;\text{unknown}\\ 1\;\text{if}\;g_{\tau_{j}}\;\text{unknown}\\ 1\;\text{if}\;g_{i}\;\text{and}\;g_{\tau_{j}}\;\text{both}\;\text{known}\;\text{and}\;g_{i}\;=\;g_{\tau_{j}}\\ 0\;\text{otherwise} \end{array}\right.$$

***Conditional report ratio***: As in the package *outbreaker2*, we allow for missing cases in transmission chains. The number of generations between cases *i* and *j*, denoted *κ _ji_*, is equal to 1 if *j* infected *i*. We define *ρ* as the conditional report ratio of the trees, which differs from the overall report ratio of an outbreak as only unreported cases within transmission chains impact the conditional report ratio. Entirely unreported clusters, or unreported cases infected earlier than the ancestor of a tree do not change the value of *ρ*. By default, the probability of observing *κ _ji_* missing generation between *i* and *j* from the conditional report ratio *p*( *κ _ji_* | *ρ*) follows an exponential distribution.

The conditional report ratio is estimated during the MCMC runs using a beta distribution prior. The two parameters of the beta prior can be changed using the variable prior_pi in create_config() (default to *Beta*(10,1)).

***Time component***: The probability of *t _i_* being the infection date of the case *i* reported at time *T _i_* depends on the distribution of the incubation period *f*. The incubation period is defined by the variable f_dens in the function outbreaker_data().

The probability that *i* was infected by *j* given their respective inferred dates of infection *t _i_* and *t _j_* is defined by the generation time of the disease *w ^κ _ji_^* ( *t _i_* – *t _j_*) (variable w_dens in outbreaker_data()), and the number of generations *κ _ji_* between *i* and *j*. The function *w ^κ _ji_^* was defined as w ^κ _ji_^ = w * w * ...* w, where * is the convolution operator applied *κ _ji_* times.

This component of the likelihood follows the framework developed in the Wallinga-Teunis method, and in *outbreaker2*.

***Spatial component***: The probability of connection between two regions *k* and *l* depends on the population sizes *m _k_* and *m _l_*, and the distance between regions *d _kl_*. Given spatial parameters *a* and *b*, *s*( *k,l*) is the probability that a case in the region *k* was infected by a case reported in *l*, and is defined using *p _kl_*, the connectivity between regions *k* and *l*:

$$s(k,l)\;=\;\frac{p_{kl}}{\Sigma_{h}p_{hl}}\;=\;\frac{F(d_{kl},b)\;*\;m_{k}^{a}\;*\;m_{l}^{c}}{\Sigma_{h}(F(d_{hl},b)\;*\;m_{h}^{a}\;*\;m_{l}^{c})}\;=\;\frac{F(d_{kl},b)\;*\;m_{k}^{a}}{\Sigma_{h}(F(d_{hl},b)\;*\;m_{h}^{a})}$$

The package comes with a built-in exponential gravity model: $F(d_{kl},a)\;=\;e^{-b*d_{kl}}$ ; or a power-law gravity model : $F(d_{kl},a)\;=\;{\left(\frac{1}{d_{kl}}\right)}^{b}$ . The exponential gravity model has been shown to be a better representation of short-distance mobility patterns ^26^; it is therefore the default option since *o2geosocial* aims at reconstructing transmission in a community or a region. The type of gravity model can be changed by setting the parameter spatial_method to “power-law”: create_config(spatial_method = "power_law"). Other mobility models can be implemented by developing modules. In the use case, we give an example on how to replace the exponential gravity by Stouffer’s rank model ^27^.

The parameters *a* and *b* are estimated during the MCMC run via posterior sampling. This requires re-computing the matrix of spatial connectivity between regions at each iteration and is time-consuming. Therefore, if either *a* or *b* is estimated, we allow for a maximum of 1 missing generation between cases ( *max*( *κ _ji_*) = 2) and only compute *s* ^1^( *k,l*) and *s* ^2^( *k,l*) for regions that could potentially be connected. By default, the prior distribution of *a* and *b* are uniform.

***Age component***: Given the age group of each case *α*_(1,.., *N*)_ and the age-stratified social contact matrix, we introduced *a ^κ _ji_^* ( *α _i_*, *α _j_*), the probability that a case aged *α _j_* infected a case aged *α _i_*. This corresponds to the proportion of contacts to *α _i_* that came from individuals of age *α _j_*. Social contact matrices provided by large scale quantitative investigations such as the POLYMOD study quantify the probability of contact between infectors and infectees of different age groups ^28^, and are imported using the R package *socialmixr* ^29^. The contact matrix used in the MCMC run is defined by the variable a_dens in outbreaker_data().

***Overall likelihood***: The overall likelihood that a case *i* was infected by the case *j* is equal to *L _i_* ( *t _i_,j,t _j_,θ*) = *log*( *f*( *t _i_ – T _i_*)) + *L _ji_* ( *t _i_,t _j_,θ*) where *f*( *t _i_ – T _i_*) is the likelihood that a case reported on *T _i_* was infected on *t _i_*, and *L _ji_* ( *t _i_,t _j_,θ*) is the log-likelihood of connection between *i* and *j* defined as:

$$L_{ji}(t_{i},t_{j},\theta )\;=\;log(\rho (\kappa_{ji}|\rho )\;*\;w^{\left(\kappa_{ji}\right)}\;(t_{i}\;-\;t_{j})\;*\;a^{\left(\kappa_{ji}\right)}\;(\alpha_{i},\alpha_{j})\;*\;G(g_{i},g_{\tau_{j}})\;*\;s^{\left(\kappa_{ji}\right)}\;(r_{i},r_{j}|a,b))$$

### Tree proposals

At every iteration of the MCMC, a set of movements is used to propose an update of the transmission trees. This update is then accepted or rejected depending on the posterior density with the proposed trees. By default, eight movements are tested at each iteration. Three of them were already part of *outbreaker2* and were not modified:

(cpp_move_t_inf() changes the infection date of the cases; cpp_move_pi()changes the conditional report ratio; cpp_move_kappa() changes the number of generations between cases). Two movements were edited to scan each transmission tree in order to prevent different genotypes from being in the same tree: (cpp_move_alpha() changes the infector; cpp_move_swap_cases() swaps infector and infectee). The remaining three are new movements:

• cpp_move_a() and cpp_move_b() change the spatial parameters *a* and *b* and update the probability of connection between regions.

• cpp_move_ancestor() changes the ancestor of the tree. An ancestor is defined as the first case of a transmission tree. For each ancestor *i*, an index case is drawn from the pool of potential infectors, while another link is randomly picked and deleted.

## Use case

### Description of the simulated data

Two simulated datasets are included in *o2geosocial*: toy_outbreak_short and toy_outbreak_long. Both are lists describing simulated outbreaks and include three elements: i) cases: a data.table with the ID, location, onset date, genotype, age group, import status, cluster, generation and infector of each case; ii) dt_regions: a data table with the ID, population, longitude and latitude of each region; iii) age_contact: a numeric matrix of the proportion of contact between age groups. Both simulations were ran using distributions of the serial interval and the latent period typically associated with measles outbreaks: the incubation period followed a gamma distribution of mean 11.5 days (standard deviation 2.24 days) ^30^; the serial interval followed a normal distribution of mean 11.7 days (standard deviation 2.0 days) ^31^.

In this use case, we analyse toy_outbreak_short. The dataset contains 75 simulated cases from different census tracts of Ohio in 2014 (variable cens_tract). The census tracts represent areas established by the Bureau of Census for analyzing populations and generally contain between 2,500 to 8,000 inhabitants. The variable cluster describes the transmission tree each case belongs to, and "generation" is equal to the number of generations between the first case of the tree ( generation = 1) and the case. 

In this use case, we reconstruct the cluster size distribution of the simulated outbreaks using different models. We then evaluate the agreement between the inferred and the reference transmission clusters in each model, and compare the results obtained with each model. Finally, we assess the geographical heterogeneity of the reconstructed transmission dynamics. We use the package *data.table* for handling data throughout as it is optimised to deal with large datasets ^32^. The methods defined in *o2geosocial* would work similarly if we had used the data.frame syntax and format.


library(o2geosocial)
## We used the data.table syntax throughout this example
library(data.table)
data("toy_outbreak_short")
# Show the first five rows
print(toy_outbreak_short$cases[1:5,])

##     ID State       Date Genotype  Cens_tract age_group import cluster
## 1: 112  Ohio 2014-01-01       B3 39005970100         6   TRUE      16
## 2:  75  Ohio 2014-01-06       D8 39139002400        11   TRUE      14
## 3: 116  Ohio 2014-01-12       B3 39101000400        11   TRUE      17
## 4: 113  Ohio 2014-01-13       B3 39005970100         6  FALSE      16
## 5: 145  Ohio 2014-01-13       D8 39117965300         8   TRUE      26
##    generation infector_ID
## 1:          1        <NA>
## 2:          1        <NA>
## 3:          1        <NA>
## 4:          2         112
## 5:          1        <NA>

# Extract dataset
dt_cases <- toy_outbreak_short[["cases"]]


**Figure 2.**
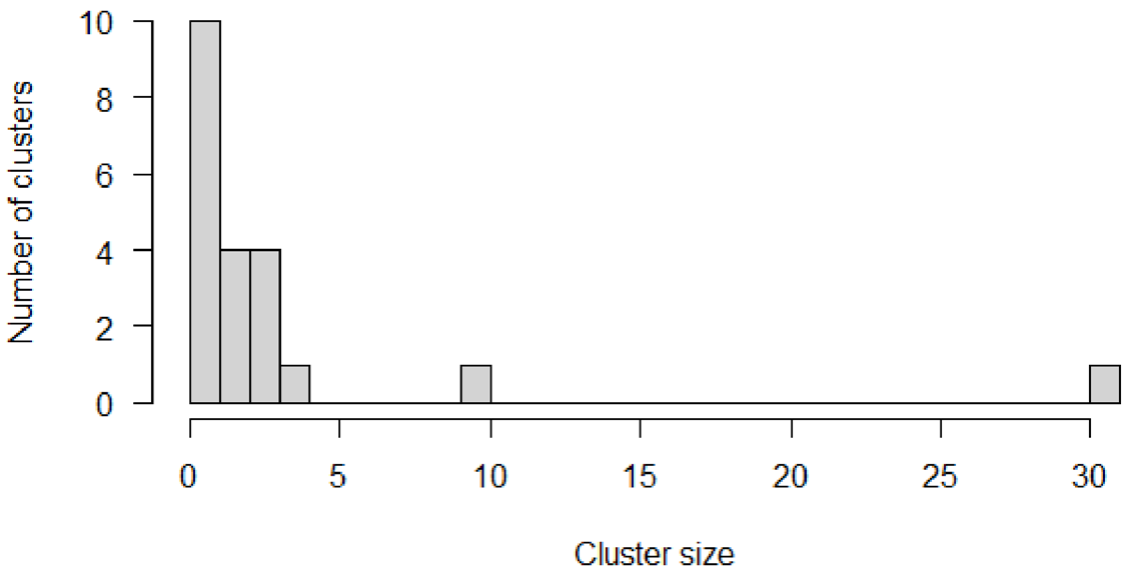
Cluster size distribution of the simulated dataset.



In the simulated data, 95% of the clusters contain less than five cases, 47.6% of the clusters are isolated (also called singletons). One larger cluster includes 31 cases ( Figure 2).

# Reference cluster size distribution
hist(table(dt_cases$cluster), breaks = 0:max(table(dt_cases$cluster)), 
      ylab = "Number of clusters", xlab = "Cluster size", main = "", las=1)


### Set up and run the models with outbreaker()

We set up the distributions the model will use to reconstruct the transmission trees. We define f_dens as the duration of the latent period, and w_dens as the serial interval. These distributions have previously been described for measles outbreaks ^30, 31, 33, 34^.



# Distribution of the latent period
f_dens <- dgamma(x = 1:100, scale = 0.43, shape = 26.83)
# Distribution of the generation time
w_dens <- dnorm(x = 1:100, mean = 11.7, sd = 2.0)


The age specific social contact patterns can be imported from the element age_contact of the list toy_outbreak_short. Alternatively, one can use the R package *socialmixr* to import a social contact matrix from the POLYMOD survey ^29^.



# From the list toy_outbreak_short  
a_dens <- toy_outbreak_short$age_contact
# Alternatively, from POLYMOD:
polymod_matrix <-
  t(socialmixr::contact_matrix(socialmixr::polymod,
                                   countries="United Kingdom",
                                   age.limits=seq(0, 70, by=5))$matrix)
polymod_matrix <-data.table::as.data.table(polymod_matrix)
# Compute the proportion of connection to each age group
a_dens <- t(t(polymod_matrix)/colSums(polymod_matrix))


Finally, the distance matrix between regions is set up from the data table dt_regions, element of toy_outbreak_short. We use the column population to set up the population vector pop_vect. We compute the distance between each region into the distance matrix dist_mat using the package *geosphere* ^35^.



# Extract all regions in the territory
dt_regions <- toy_outbreak_short[["dt_regions"]]
# Extract the population vector
pop_vect <- dt_regions$population
# Create the matrices of coordinates for each region (one "from"; one "to")
mat_dist_from <- matrix(c(rep(dt_regions$long, nrow(dt_regions)),
                             rep(dt_regions$lat, nrow(dt_regions))), ncol = 2)
mat_dist_to <- matrix(c(rep(dt_regions$long, each = nrow(dt_regions)), 
                           rep(dt_regions$lat, each = nrow(dt_regions))),
                         ncol = 2)
# Compute all the distances between the two matrices
all_dist <- geosphere::distGeo(mat_dist_from, mat_dist_to)
# Compile into a distance matrix
dist_mat <- matrix(all_dist/1000, nrow = nrow(dt_regions))
# Rename the matrix columns and rows, and the population vector
names(pop_vect) <- rownames(dist_mat) <- colnames(dist_mat) <-
  dt_regions$region


We create the lists data, config, moves, likelihoods and priors to run the main function of the package. In this example, we use the default parameters to build moves, likelihoods and priors. The list data contains the distributions f_dens and w_dens, the population vector and the distance matrix, along with the onset dates, age group, location and genotype of the cases.

Routinely collected surveillance data can include information on the importation status of the cases. In order to investigate the impact of using prior information on the importation status of the cases on cluster reconstruction, we implement two different models: in out1 the import status is inferred by the model, whereas in out2 it is set as an input parameter of the model, which only estimates who infected whom.

The first short run in out1 is run with 10,000 iterations to find the minimum number of importations, and the main run lasts for 20,000 iterations in both models. As the import status of the cases is inferred in out1, we have to set a threshold to quantify what is an unlikely likelihood of transmission between cases. We use a relative outlier threshold at 0.9, which means that the threshold will be the 9 ^*th*^ decile of the negative log-likelihoods *L _i_* ( *t _i_,j,t _j_,θ*) in every sample.


# Set movement, likelihood and prior lists to default
moves <- custom_moves()
likelihoods <- custom_likelihoods()
priors <- custom_priors()
# Data and config, model 1
data1 <- outbreaker_data(dates = dt_cases$Date, #Onset dates
                            age_group = dt_cases$age_group, #Age group
                            region = dt_cases$Cens_tract, #Location
                            genotype = dt_cases$Genotype, #Genotype
                            w_dens = w_dens, #Serial interval
                            f_dens = f_dens, #Latent period
                            a_dens = a_dens, #Age stratified contact matrix
                            population = pop_vect, #Population 
                            distance = dist_mat #Distance matrix
)
config1 <- create_config(data = data1, 
                            n_iter = 20000, #Iteration number: main run
                            n_iter_import = 10000, #Iteration number: short run
                            burnin = 5000, #burnin period: first run
                            outlier_relative = T, #Absolute / relative threshold 
                            outlier_threshold = 0.9 #Value of the threshold
)
# Run model 1
out1 <- outbreaker(data = data1, config = config1, moves = moves, 
                     priors = priors, likelihoods = likelihoods)
# Set data and config for model 2
data2 <- outbreaker_data(dates = dt_cases$Date, 
                            age_group = dt_cases$age_group,
                            region = dt_cases$Cens_tract,
                            genotype = dt_cases$Genotype, w_dens = w_dens, 
                            f_dens = f_dens, a_dens = a_dens,
                            population = pop_vect, distance = dist_mat,
                            import = dt_cases$import #Import status of the cases
)
config2 <- create_config(data = data2, 
                            find_import = FALSE, # No inference of import status
                            n_iter = 20000, 
                            sample_every = 50, # 1 in 50 iterations is kept
                            burnin = 5000)
# Run model 2
out2 <- outbreaker(data = data2, config = config2, moves = moves, 
                      priors = priors, likelihoods = likelihoods)


The data frames out1 and out2 contain the posterior density, likelihood, and prior density of the trees generated at every iteration, along with the values of the spatial parameters a and b, the conditional report ratio pi, and the index, estimated infection date and number of generations for each case.

### Compare inferred and reference clusters

The function summary prints a summary of the data frame generated by outbreaker(). It contains a list with the number of steps, the distributions of the posterior, likelihood and priors, the parameter distributions, the most likely infector and the probability of being an import for each case, and the cluster size distribution.


# Summary parameters a and b, removing the burnin-period
#Model 1
print(summary(out1, burnin = 5000)$a) 

##    Min. 1st Qu.  Median    Mean 3rd Qu.    Max. 
##  0.2011  0.5920  0.8398  0.8569  1.1183  1.4965

print(summary(out1, burnin = 5000)$b)

##    Min. 1st Qu.  Median    Mean 3rd Qu.    Max. 
## 0.07081 0.09187 0.09943 0.09905 0.10634 0.13570

# Model 2
print(summary(out2, burnin = 5000)$a)

##    Min. 1st Qu.  Median    Mean 3rd Qu.    Max. 
##  0.2174  0.6710  0.9522  0.9152  1.2084  1.4983

print(summary(out2, burnin = 5000)$b)

##    Min. 1st Qu.  Median    Mean 3rd Qu.    Max. 
## 0.09641 0.12084 0.12960 0.13079 0.14053 0.19137


In order to compare the reconstructed clusters to the data in each model, we plot the median inferred cluster size distribution in out1 and out2 and the credible intervals. First, we group together clusters of similar sizes by defining the breaks of each group in the vector group_cluster. In this example, we defined the size categories as 1; 2; 3 – 4; 5 – 9; 10 – 15; 15 – 40 and 40 + cases. The inferred cluster size distributions are shown in the element cluster from the output of summary(out1), and are aggregated using the input variable group_cluster.


# We create groups of cluster size: initialise the breaks for each group
group_cluster <- c(1, 2, 3, 5, 10, 15, 40, 100) - 1
# Reference data: h$counts
h <- hist(table(dt_cases$cluster), breaks = group_cluster, plot = FALSE)

# Grouped cluster size distribution in each run
clust_infer1 <- summary(out1, group_cluster = group_cluster, 
                           burnin = 5000)$cluster
clust_infer2 <- summary(out2, group_cluster = group_cluster, 
                           burnin = 5000)$cluster
# Merge inferred and reference cluster size distributions into one matrix
clust_size_matrix <- rbind(clust_infer1["Median",], clust_infer2["Median",],
                           h$counts)



**Figure 3.**
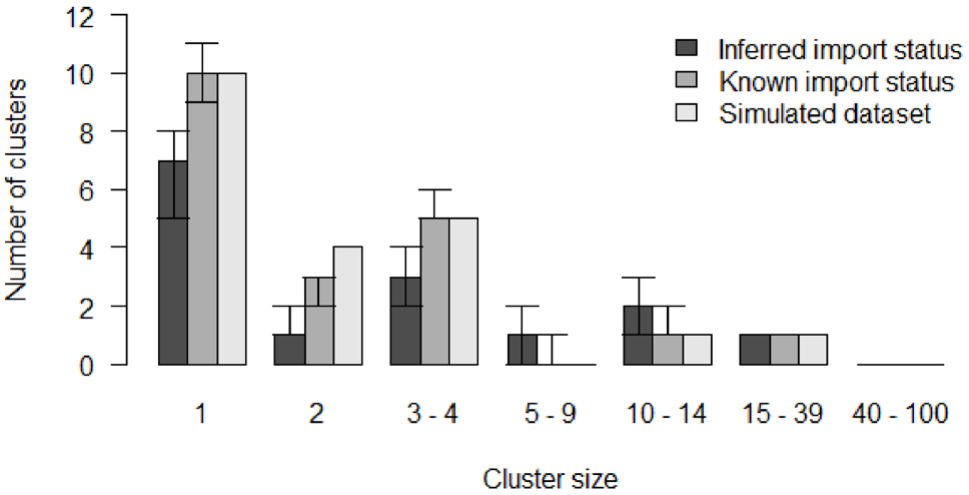
Comparison of inferred cluster size distribution in both models with the reference data.


The number of isolated cases in the inferred trees in out1 is lower than in the data ( Figure 3). We can therefore conclude that when the import status of the cases was inferred, the model underestimated the number of clusters and tended to link together unrelated cases. The cluster size distribution when the import status of the cases is inferred depends on the likelihood threshold set in outlier_threshold and outlier_relative. Using different values of *λ* would impact the cluster size distribution in out1. Conversely, the cluster size distribution in out2 is very similar to the data ( Figure 3).

# Histogram of the inferred and reference cluster size distributions 
b <- barplot(clust_size_matrix, names.arg = colnames(clust_infer1), las=1,
              ylab = "Number of clusters", xlab = "Cluster size", main = "", 
              beside = T, ylim = c(0, max(c(clust_infer1, clust_infer2))))
# Add the 50% CI
arrows(b[1,], clust_infer1["1st Qu.",], b[1,], clust_infer1["3rd Qu.",], 
        angle = 90, code = 3, length = 0.1)
arrows(b[2,], clust_infer2["1st Qu.",], b[2,], clust_infer2["3rd Qu.",], 
        angle = 90, code = 3, length = 0.1)
# Add legend
legend("topright", fill = grey.colors(3), bty = "n",
        legend = c("Inferred import status", 
                    "Known import status", "Simulated dataset"))


We investigate the reconstructed transmission trees to ensure the index assigned to each case is in agreement with the reference dataset. To do so, we write two functions: in index_infer we compute the proportion of iterations where the inferred index of each case matches their actual index (perfect match); in index_clust we compute the proportion of iterations where the inferred index is from the same reference cluster as the actual index (close match).


#' Title: Compute the proportion of iterations in the outbreaker() output 
#` where the inferred index matches the actual index in dt_cases
#'
#' @param dt_cases: reference dataset
#' @param out: Matrix output of outbreaker()
#' @param burnin: Numeric, length of the burnin phase
#'
#' @return Numeric vector showing the proportion of iterations pointing to
#' the correct index case
index_infer <- function(dt_cases, out, burnin){
  ## Generate the data frame listing every infector:
  # Select rows above burnin, and columns describing who infected whom
  out_index <- out[out$step > burnin, grep("alpha", colnames(out))]
  # ID of each infector
  ID_index <- matrix(dt_cases[unlist(out_index), ID], ncol = nrow(dt_cases))
  # Match inferred (ID_index) and actual infector (column infector_ID)
  match_infer_data <- t(ID_index) == dt_cases$infector_ID
  # If a case is rightly inferred as an ancestor, set match to TRUE
  match_infer_data[is.na(t(ID_index)) & is.na(dt_cases$infector_ID)] <- TRUE
  prop_correct <- rowSums(match_infer_data, na.rm = T)/ncol(match_infer_data)
  
  return(prop_correct)
}




# Same as index_infer, except it returns the proportion of inferred indexes
# who are in the same reference cluster as the case
index_clust <- function(dt_cases, out, burnin){
  ## Generate the data frame listing every infector:
  # Select rows above burnin, and columns describing who infected whom
  out_index <- out[out$step > burnin, grep("alpha", colnames(out))]
  # cluster of each infector
  clust_index <- matrix(dt_cases[unlist(out_index), cluster], 
                           ncol = nrow(dt_cases))
  # Match inferred (cluster_index) and actual cluster (column cluster)
  match_infer_data <- t(clust_index) == dt_cases$cluster
  # Exclude ancestors
  match_infer_data <- match_infer_data[!is.na(dt_cases$infector_ID),]
  
  prop_correct <- rowSums(match_infer_data, na.rm = T)/ncol(match_infer_data)
  
  return(prop_correct)
}
# Run index_infer for each model
index_infer1 <- index_infer(dt_cases = dt_cases, out = out1, burnin = 5000)
index_infer2 <- index_infer(dt_cases = dt_cases, out = out2, burnin = 5000)
# Run index_clust for each model
index_clust1 <- index_clust(dt_cases = dt_cases, out = out1, burnin = 5000)
index_clust2 <- index_clust(dt_cases = dt_cases, out = out2, burnin = 5000)



**Figure 4.**
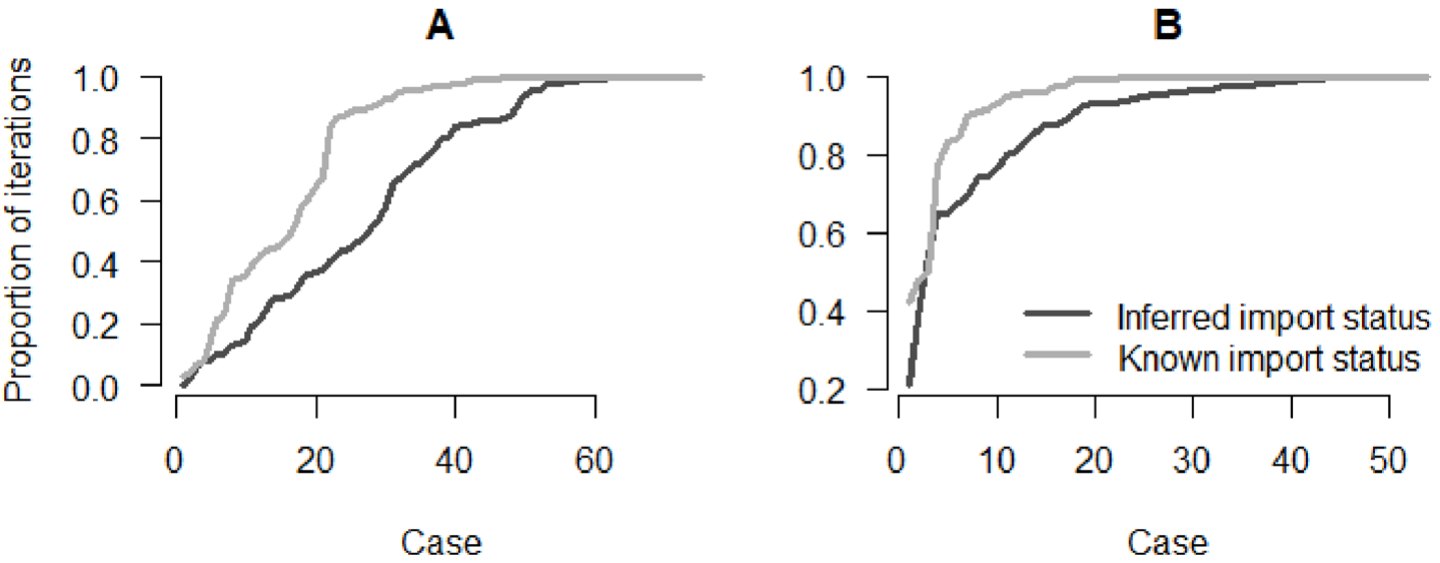
Panel **A**: Proportion of iterations with the correct index for each case; Panel **B**: Proportion of iterations where the index is from the correct cluster.


Figure 4 shows that the proportion of perfect and close match for most cases is lower in out1, which indicates that inferring the import status reduced the accuracy of the inference. Using previous investigations into the travel history of cases is key to improve the reconstruction of transmission history.

# Plot the sorted proportion in each model
par(bty = "n", mfrow = c(1, 2), mar = c(5,4,2,0), oma = c(0, 0, 0, 0))
# Panel A: Perfect match
plot(sort(index_infer1), type = "l", ylab = "Proportion of iterations", xlab = "Case", 
      main =  "A", las=1, col = grey.colors(3)[1], lwd = 3)
lines(sort(index_infer2), col = grey.colors(3)[2], lwd = 3)

# Panel B: Close match
plot(sort(index_clust1), type = "l", xlab = "Case", ylab = "", 
      main =  "B", las=1, col = grey.colors(3)[1], lwd = 3)
lines(sort(index_clust2), col = grey.colors(3)[2], lwd = 3)
legend("bottomright", col = grey.colors(3)[1:2], lwd = 3, bty = "n",
        legend = c("Inferred import status","Known import status"))


We now investigate the geographical distribution of the importations, and the average number of secondary cases per region in out1 and out2. The maps are generated using the package *ggplot2* ^36^.

First, we retrieve the boundary files of the census tracts in Ohio to generate the background of the maps using the package *tigris* ^37^. We import them in a format compatible with the package sf and create one background map for each model.



library(ggplot2)
# Read the shapefile and create one map for each model
map1 <- tigris::tracts(state = "Ohio", class = "sf", progress_bar = FALSE)
map1$INTPTLON <- as.numeric(map1$INTPTLON)
map1$INTPTLAT <- as.numeric(map1$INTPTLAT)
map2 <- map1
map1$model <- "Model 1"
map2$model <- "Model 2"


We are interested in two outputs of the models: i) the number of imports per region, in order to highlight regions where importations of cases are most likely, and ii) the geographical distribution of the number of secondary cases per case, which gives insight into the areas most vulnerable to the spread of the disease.

***Number of imports per region***: The element tree of summary(out1) contains the most likely infector, the proportion of iterations where the index is the most likely infector and the median number of generations between the two cases, the most likely infection date and the chances of being an import for each case. We add two columns to dt_cases showing the probablity of being an import in out1 and out2 for each case. As the import status is not inferred in out2, prop_import2 is a binary value, and is equal to dt_cases$import.



# Add the proportion of iterations in model 1 where each case is an import
dt_cases[, prop_import1 := summary(out1, burnin = 5000)$tree$import]
# Add the proportion of iterations in model 2 where each case is an import
dt_cases[, prop_import2 := summary(out2, burnin = 5000)$tree$import]


We generate the number of imports per region in each model (vectors prop_reg1 and prop_reg2) and add it to the matrices describing the maps.



# Number of imports per region in model 1
prop_reg1 <- dt_cases[, .(prop_per_reg = sum(prop_import1)), 
                         by = Cens_tract]$prop_per_reg
# Number of imports per region in model 2
prop_reg2 <- dt_cases[, .(prop_per_reg = sum(prop_import2 )), 
                         by = Cens_tract]$prop_per_reg
names(prop_reg1) <- names(prop_reg2) <- unique(dt_cases$Cens_tract)

# Add the number of imports in each region to the maps
map1$prop_reg <- prop_reg1[as.character(map1$GEOID)]
map2$prop_reg <- prop_reg2[as.character(map2$GEOID)]


**Figure 5.**
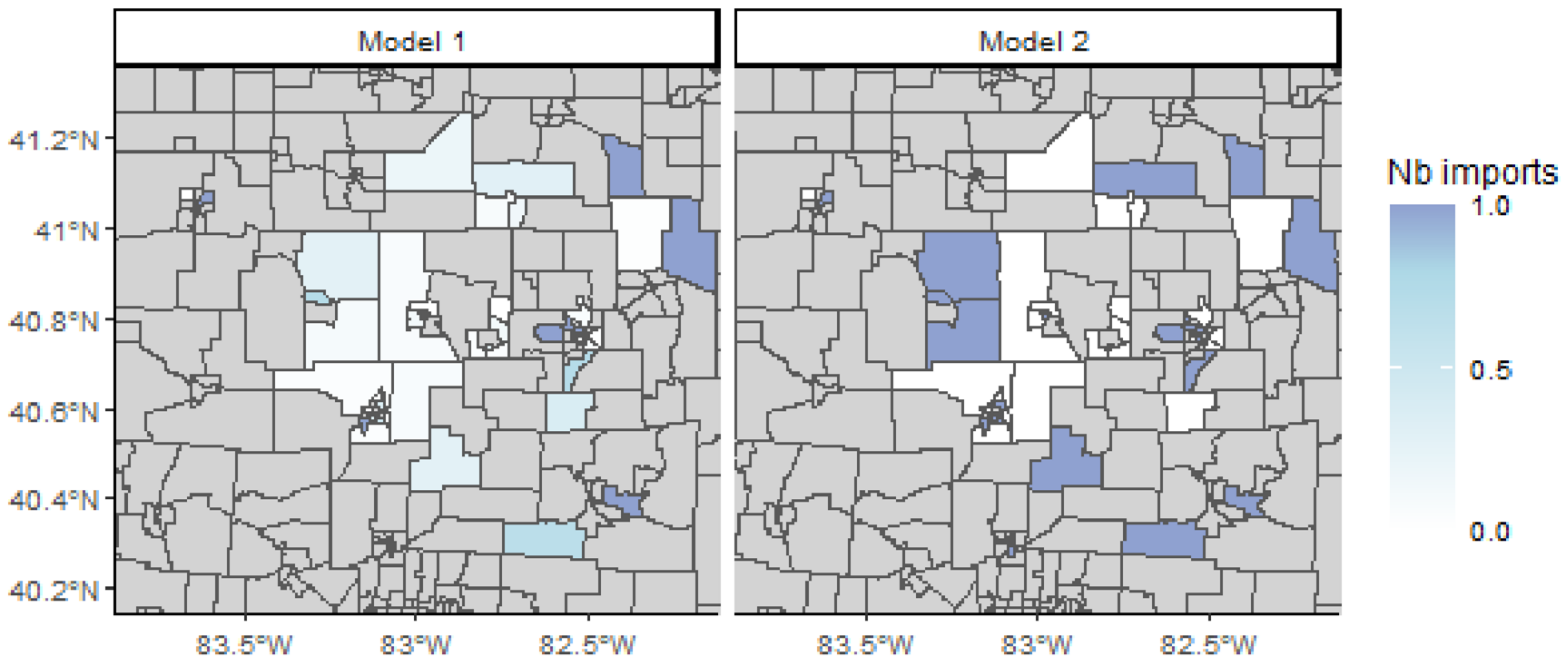
Average number of imported cases per census tract, regions where no case was reported are shown in grey.



We plot the number of imports per region in each model ( Figure 5). The right panel ( out2) shows the geographical distribution of importations in the data. We observe discrepancies between the two panels. In out1, the inferred number of importations in the central areas is much lower than in the reference data. These maps highlight the uncertainty added when the import status of each case is inferred.

# Merge maps
maps <- rbind(map1, map2)
# Crop map to area of interest
lim_lon <- c(-84, -82)
lim_lat <- c(40, 41.5)
maps <- maps[maps$INTPTLON > lim_lon[1] & maps$INTPTLON < lim_lon[2] & 
                 maps$INTPTLAT > lim_lat[1] & maps$INTPTLAT < lim_lat[2],]

# Plot: number of imports per region, two panels
ggplot(maps) +  geom_sf(aes(fill = prop_reg))+ facet_grid(~model)+     
  scale_fill_gradient2(na.value = "lightgrey", midpoint = 0.8, 
                          breaks = c(0, 0.5, 1, 1.5), name = "Nb imports",
                          low = "white", mid = "lightblue", high = "darkblue") + 
  coord_sf(xlim = c(-83.8, -82.2), ylim = c(40.2, 41.3)) +
  theme_classic(base_size = 9)


***Average number of secondary cases per region***: In this section, we map the number of secondary cases per case in each region to identify which regions were associated with higher levels of transmission. We define the function n_sec_per_reg to compute the average number of secondary cases per case and aggregate it per region. We then extract the median number of secondary cases per case in each region.



#' Title: Compute the number of secondary cases per case in each region
#'
#' @param dt_cases: reference dataset
#' @param out: Matrix output of outbreaker()
#' @param burnin: Numeric, length of the burnin phase
#'
#' @return A numeric matrix: the first column is the census tract ID, the
#' other columns show the number of secondary cases per case. Each row 
#' corresponds to a different iteration.
n_sec_per_reg <- function(dt_cases, out, burnin){
  ## Number of secondary cases per case
  n_sec <- apply(out[out$step > burnin, grep("alpha", colnames(out))], 1, 
                   function(X){
                     X <- factor(X, 1:length(X))
                     return(table(X))})
  ## Aggregate by region
  tot_n_sec_reg <- aggregate(n_sec, list(dt_cases$Cens_tract), sum)
  ## Divide by the number of cases in each region
  tot_n_sec_reg <- cbind(tot_n_sec_reg[, 1], 
                            tot_n_sec_reg[, -1] / table(dt_cases$Cens_tract))
  return(tot_n_sec_reg)
}
## Generate the number of secondary cases per case in each region
n_sec_tot1 <- n_sec_per_reg(dt_cases = dt_cases, out = out1, burnin = 5000)
n_sec_tot2 <- n_sec_per_reg(dt_cases = dt_cases, out = out2, burnin = 5000)
## Compute the median in each model
n_sec1 <- apply(n_sec_tot1[,-1], 1, median)
n_sec2 <- apply(n_sec_tot2[,-1], 1, median)
names(n_sec1) <- names(n_sec2) <- unique(dt_cases$Cens_tract)
## Add to the matrices describing the maps
map1$n_sec <- as.numeric(n_sec1[as.character(map1$GEOID)])
map2$n_sec <- as.numeric(n_sec2[as.character(map2$GEOID)])



**Figure 6.**
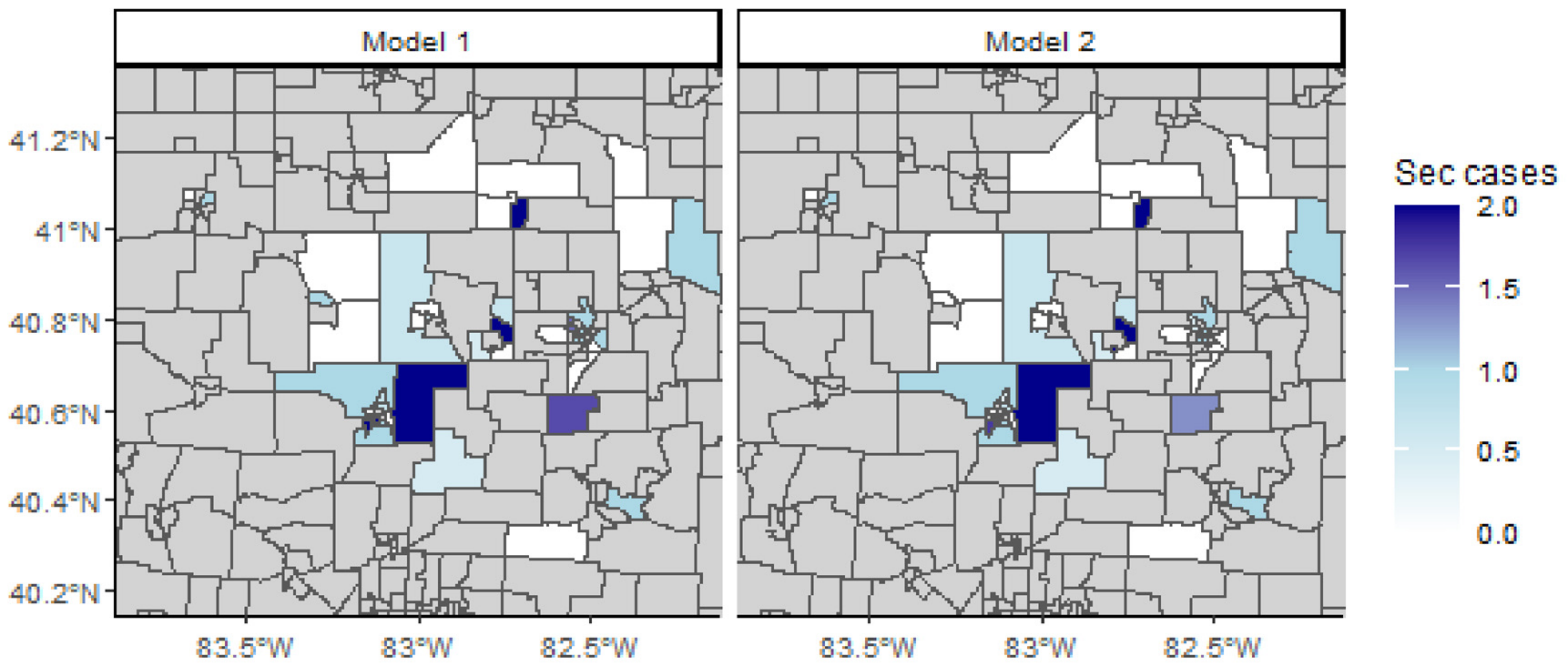
Median number of secondary transmission per case in each census tract.



We now plot the geographical distribution of the median number of secondary cases in each region ( Figure 6). Despite minor discrepancies, the maps generated by the two models are similar. Both show an important spatial heterogeneity. The eastern and central areas are associated with higher numbers of secondary cases. If we change the vectors n_sec1 and n_sec2 to plot different deciles, we show the dispersion of the number of secondary cases in the different iterations of the models.

# Merge maps
maps_n_sec <- rbind(map1, map2)
# Crop map to area of interest
lim_lon <- c(-84, -82)
lim_lat <- c(40, 41.5)
maps_n_sec <- maps_n_sec[maps_n_sec$INTPTLON > lim_lon[1] &
                               maps_n_sec$INTPTLON < lim_lon[2] &
                               maps_n_sec$INTPTLAT > lim_lat[1] & 
                               maps_n_sec$INTPTLAT < lim_lat[2],]

# Plot the geographical distribution of the number of secondary cases
ggplot(maps_n_sec) +  geom_sf(aes(fill = n_sec)) + facet_grid(~model)  +     
  scale_fill_gradient2(na.value = "lightgrey", mid = "lightblue",
                          low = "white", midpoint = 1, high = "darkblue",
                          breaks = seq(0, 5, 0.5),name = "Sec cases") +
  coord_sf(xlim = c(-83.8, -82.2), ylim = c(40.2, 41.3)) +
  theme_classic(base_size = 9)


### Customise the likelihood, prior and movement lists: the Stouffer’s rank model

In the previous example, we ran and evaluated two different models to reconstruct transmission clusters from simulated surveillance data, and highlighted the spatial heterogeneity of measles transmission in the region. These models were run using the default likelihood, prior and movement functions. Now we develop a third model, where the spatial connection between regions is based on the Stouffer’s rank method ^27^.

In the Stouffer’s rank method, the absolute distance is not used to compute the probability of connection between regions. The connectivity between the regions *k* and *l* only depends on the summed population of all the regions closer to *l* than *k*. If we define this collection of regions *Ω _k,l_* = { *i*: 0 ≤ *d*( *i,l*) ≤ *d*( *k,l*)}, Stouffer’s distance is then $p_{kl}\;=\;m_{l}^{c}\;*\;{\left(\frac{m_{k}}{\sum_{i\in \Omega_{k,l}}m_{i}}\right)}^{a}$ . From this, we deduce the probability that a case from region *l* was infected by a case from region *k*.

$$s(k,l)\;=\;\frac{p_{kl}}{\Sigma_{h}p_{hl}}\;=\;\frac{{\left(\frac{m_{k}}{\sum_{i\in \Omega_{k,l}}m_{i}}\right)}^{a}}{\Sigma_{h}{\left(\frac{m_{h}}{\sum_{i\in \Omega_{h,l}}m_{i}}\right)}^{a}}$$

This model is similar to the power-law gravity model with two main differences: i) each cell of the distance matrix should be equal to ${\text{Σ}}_{i\in \Omega_{k,l}}\;m_{i}$ , and ii) only one spatial parameter *a* is estimated. First, we create the distance matrix associated with Stouffer’s rank:


# For every column of the distance matrix, use the cumulative sum of the 
# population vector ordered by the distance. Remove the values where 
# the distance between the regions is above gamma
dist_mat_stouffer <- apply(dist_mat, 2, function(X){
  pop_X <- cumsum(pop_vect[order(X)])
  omega_X <- pop_X[names(X)]
  # omega_X is set to -1 if the distance between two regions is above gamma
  omega_X[X > config1$gamma] <- -1
  return(omega_X)
})
# The new value of gamma is equal to the maximum of dist_mat_stouffer + 1
gamma <- max(dist_mat_stouffer) + 1
# The values previously set to -1 are now set to the new value of gamma
dist_mat_stouffer[dist_mat_stouffer == -1] <- max(dist_mat_stouffer) * 2


Secondly, since the connectivity matrix in the Stouffer’s rank model is only computed from one spatial parameter, we write a new movement function cpp_stouffer to estimate it. The formula of the Stouffer’s rank connectivity matrix is similar to the power law gravity models. Therefore, cpp_stouffer is similar to the default movement cpp_move_a, and uses the same function to compute the probability matrix (cpp_log_like()). This function is written with the package *Rcpp*, and is sourced using the function Rcpp::sourceCpp ^22^.



// [[Rcpp::depends(o2geosocial)]]
#include <Rcpp.h>
#include <Rmath.h>
#include <o2geosocial.h>
// This function is used to estimate new values of the spatial parameter.
// It is based on the structure as cpp_move_a in o2geosocial,
// [[Rcpp::export()]]
Rcpp::List cpp_stouffer(Rcpp::List param, Rcpp::List data, Rcpp::List config,
                           Rcpp::RObject custom_ll, Rcpp::RObject custom_prior){
  // Import parameters
  Rcpp::List new_param = clone(param);
  double gamma = config["gamma"];
  int max_kappa = config["max_kappa"];
  Rcpp::List new_log_s_dens = new_param["log_s_dens"];
  Rcpp::NumericMatrix dist = data["distance"], probs = new_log_s_dens[0];
  Rcpp::NumericMatrix ances = data["can_be_ances_reg"];
  Rcpp::NumericVector pop = data["population"], limits = config["prior_a"];
  // Size of the probability matrix
  int nb_cases = pow(probs.size(), 0.5);
  // Draw new value of a
  Rcpp::NumericVector new_a = new_param["a"];
  double sd_a = static_cast<double>(config["sd_a"]);
  double old_logpost = 0.0, new_logpost = 0.0, p_accept = 0.0;
  // proposal (normal distribution with SD: config$sd_a)
  new_a[0] += R::rnorm(0.0, sd_a); // new proposed value
  if(new_a[0] < limits[0] || new_a[0] > limits[1])return param;
  // Generate new probability matrix
  new_param["log_s_dens"] = 
    o2geosocial::cpp_log_like(pop, dist, ances, new_a[0], new_a[0], 
                                 max_kappa, gamma, "power-law", nb_cases);
  // Compare old and new likelihood values
  old_logpost = o2geosocial::cpp_ll_space(data, config, param, 
                                               R_NilValue, custom_ll);
  new_logpost = o2geosocial::cpp_ll_space(data, config, new_param,
                                               R_NilValue, custom_ll);
  // Add prior values
  old_logpost += o2geosocial::cpp_prior_a(param, config, custom_prior);
  new_logpost += o2geosocial::cpp_prior_a(new_param, config, custom_prior);
  // Accept or reject proposal
  p_accept = exp(new_logpost - old_logpost);
  if (p_accept < unif_rand()) return param;
  return new_param;
}


We modify the element a of the list of movements used in the last model. We set up the lists data and config using dist_mat_stouffer as the distance matrix. Since there is only one spatial parameter in this model, we set the parameter move_b to FALSE in create_config(), and we set the prior of b to the null function f_null.



# Edit the lists of movements and priors
moves3 <- custom_moves(a = cpp_stouffer)
# Define null function
f_null <- function(param) {
  return(0.0)
}
priors3 <- custom_priors(b = f_null)

# Set data and config lists
data3 <- outbreaker_data(dates = dt_cases$Date, #Onset dates
                            age_group = dt_cases$age_group, #Age group
                            region = dt_cases$Cens_tract, #Location
                            genotype = dt_cases$Genotype, #Genotype
                            w_dens = w_dens, #Serial interval
                            f_dens = f_dens, #Latent period
                            a_dens = a_dens, #Age stratified contact matrix
                            population = pop_vect, #Population 
                            distance = dist_mat_stouffer #Distance matrix

)
config3 <- create_config(data = data3, 
                            gamma = gamma,
                            init_b = 0, move_b = FALSE, # b is not estimated
                            n_iter = 20000, #Iteration number: main run
                            n_iter_import = 10000, #Iteration number: short run
                            burnin = 5000, #burnin period: first run
                            outlier_relative = T, #Absolute / relative threshold
                            outlier_threshold = 0.9 #Value of the threshold

)
# Run the model using the Stouffer's rank method
out_stouffer <- outbreaker(data = data3, config = config3, moves = moves3, 
                               priors = priors3, likelihoods = likelihoods)


**Figure 7.**
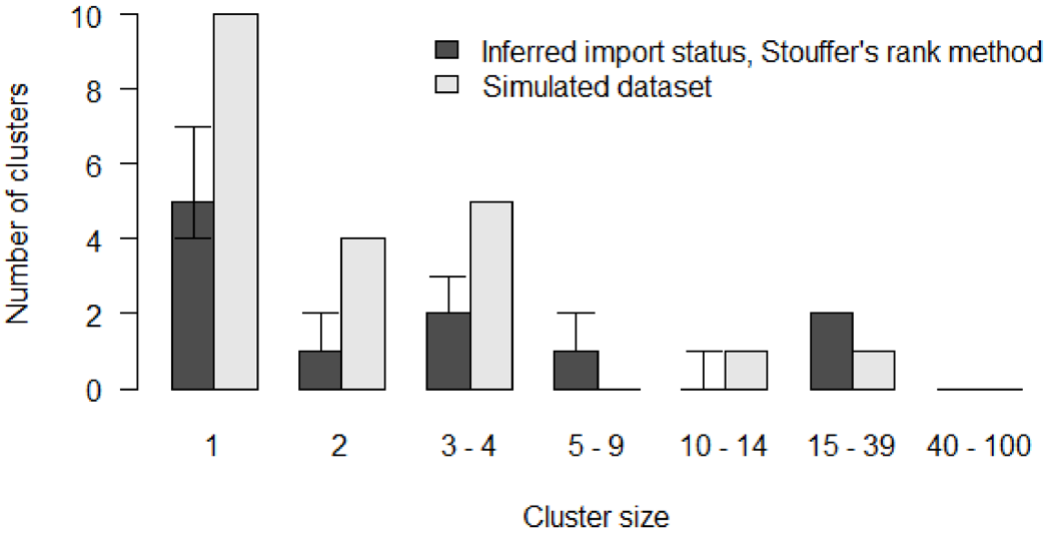
Comparison of inferred cluster size distribution with the reference data.


We plot the inferred cluster size distribution and compare it to the reference data ( Figure 7). We observe discrepancies between the inferred distribution and the data: the model over-estimates the number of clusters containing more than 15 cases and underestimates the number of small clusters and isolated individuals.

# Grouped cluster size distribution in the Stouffer's rank model
clust_infer_stouf <- summary(out_stouffer, burnin = 5000, 
                                group_cluster = group_cluster)$cluster
# Merge inferred and reference cluster size distributions
clust_size_matrix <- rbind(clust_infer_stouf["Median",], h$counts) 
# Plot the two distributions
b <- barplot(clust_size_matrix, names.arg = colnames(clust_infer_stouf), 
              beside = T, ylab = "Number of clusters", xlab = "Cluster size", 
              main = "", las = 1)
# Add CIs
arrows(b[1,], clust_infer_stouf["1st Qu.",], b[1,], 
        clust_infer_stouf["3rd Qu.",], angle = 90, code = 3, length = 0.1)
legend("topright", fill = grey.colors(2), bty = "n",
        legend = c("Inferred import status, Stouffer's rank method", 
                    "Simulated dataset"))


**Figure 8.**
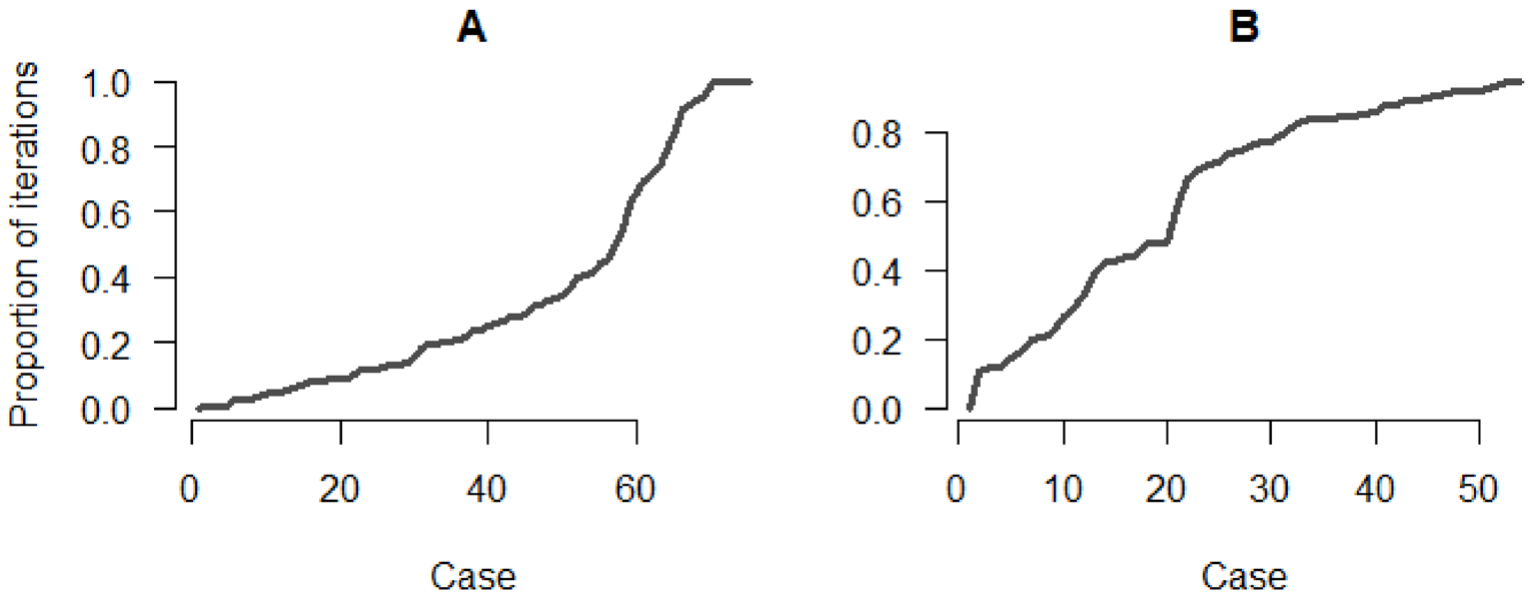
Panel **A**: Proportion of iterations with the correct index for each case; Panel **B**: Proportion of iterations where the index is from the correct cluster.

Finally, we plot the proportion of perfect and close matches for each case ( Figure 8). We observe that the fit obtained with the Stouffer’s rank method is consistently worse than the first two models. The Stouffer’s rank method did not improve the agreement between the inferred trees and the reference data.

The simulated data used in the study were generated using an exponential gravity model, which explains why introducing the Stouffer’s rank method did not improve the inference. This is not representative of the performance of each mobility model at reconstructing actual transmission clusters.


# Generate the proportion of perfect and close match for each case in out3
index_infer_stouf <- index_infer(dt_cases = dt_cases, out = out_stouffer, 
                                     burnin = 5000)
index_clust_stouf <- index_clust(dt_cases = dt_cases, out = out_stouffer, 
                                     burnin = 5000)
# Plot the sorted proportion in each model
par(bty = "n", mfrow = c(1, 2), mar = c(5,4,2,0), oma = c(0, 0, 0, 0))
# Panel A: Perfect match
plot(sort(index_infer_stouf), main = "A", col = grey.colors(2)[1], lwd = 3,
     xlab = "Case", ylab = "Proportion of iterations", type = "l", las=1)
# Panel B: Close match
plot(sort(index_clust_stouf), type = "l", ylab = "", xlab = "Case", 
     main =  "B", las=1, col = grey.colors(2)[1], lwd = 3)


## Conclusion

The R package *o2geosocial* is a new tool for data analysis building upon the framework developed in *outbreaker2*. It uses routinely collected surveillance data to reconstruct transmission networks. It can be used on a broad range of diseases where genetic sequencing is not common, or informative. For instance, it has been applied on national measles surveillance data to reconstruct the cluster size distribution of outbreaks in the United States between 2001 and 2016 ^4^. In this study, we presented an application on a simulated dataset using detailed geographic information on the location of cases.

We implemented several models to reconstruct the cluster size distribution of the simulated outbreak. Although each model was able to capture the overall dynamics of transmission, we observed discrepancies between the reference data and the reconstructed cluster size distribution for models where the importation status of the cases was inferred. These discrepancies are linked to the threshold set to define what is considered an unlikely connection. A looser threshold may lead to unrelated cases being connected and a lower number of inferred imports, whereas a stricter threshold increases the number of short transmission chains. Therefore, the use of epidemiological information describing importation status improves the accuracy of the transmission cluster reconstruction in *o2geosocial*. In case of incomplete epidemiological information, the user can set the importation status for some of the cases, and the others would be inferred. These results highlight that epidemiological investigations are crucial to improve our ability to reconstruct transmission events, particularly when unrelated importations happen concurrently.

The method described in this paper does not account for long-distance transmission, as transmission events are impossible in *o2geosocial* when the distance between regions is above the parameter gamma. In case of long-distance transmission, the infected case would be considered as a new importation. Nevertheless, this limitation is not critical since *o2geosocial* was designed to identify areas most susceptible to local transmission, *i.e.* regions where importations were likely to lead to local outbreaks.

The analyses presented in this paper were ran on simulated data, which partly explains the very close match between the inferred and reference cluster size distribution. Indeed, the distributions of the incubation period and serial interval used to generate the simulations were the same as the ones used for cluster inference. Using imprecise or inaccurate distributions can lead to biases in the reconstruction of the transmission trees.

We also showed how the model could be edited to implement different mobility models. Describing human mobility during infectious diseases outbreaks is challenging, and the performance of the models depends on the setting ^26, 38– 40^. We encourage the development of extensions of *o2geosocial* to study a wide range of pathogens and settings where sequence data are not informative. We hope that wider use of *o2geosocial* can help maximise the information brought by routinely collected data and epidemiological investigations, in order to improve our understanding of outbreak dynamics.

## Data availability

Zenodo: o2geosocial. https://doi.org/10.5281/zenodo.4317440 ^23^.

This project contains the following underlying data:

- alxsrobert/o2geosocial-v1.0.1.zip (data folder; simulated data generated from measles virus incubation period and serial interval)

Data are available under the terms of the Open Source Initiative MIT license.

## Software availability

**Software available from:** https://CRAN.R-project.org/package=o2geosocial.

**Source code available from:** https://github.com/alxsrobert/o2geosocial.

**Archived source code at time of publication:** https://doi.org/10.5281/zenodo.4317440 ^23^.

**License:** MIT license.
