# Supplementary material for: o2geosocial: Reconstructing who-infected-whom from routinely collected surveillance data
Source: F1000Res. 2021 Jun 16;10:31. Originally published 2021 Jan 18. [Version 2] doi: 10.12688/f1000research.28073.2 (PMC10044721; doi:10.12688/f1000research.28073.2)
Supplement: Peer review history

# Peer review: Reviewer response for version 2

**DOI:** 10.5256/f1000research.57311.r165883

**Published:** 2023-03-22

**Authors:** Gerry Tonkin Hill

**Affiliations:** 1 Universitetet i Oslo, Oslo, Oslo, Norway

Robert *et al.* present an R package for inferring transmission chains using routinely collected surveillance data. The package is specifically designed for scenarios where integrating genomic data into the likelihood function is not as effective, such as for pathogens with extremely low mutation rates. The manuscript addresses an important problem and the R package is likely to be useful to both researchers and public health laboratories.

 There are a few areas that I think the authors could expand on.

- The simulated outbreak consisted primarily of very small clusters with ~47% involving only a single isolate. Smaller clusters considerably simplify the problem of inferring transmission chains. It would be good if the authors could investigate how the accuracy of the approach changes with larger clusters. This could be achieved with additional simulations or by adjusting/adding to Figure 4 to indicate how cluster size impacted these results.
- It would be useful to include an additional figure to Figure 4 that clearly indicated the number of correctly inferred infector/infectee pairs if a particular approach, such as taking the MAP estimate, was taken. Perhaps a plot similar to the ‘alpha’ plot of *outbreaker2* could be used. 
- It was not clear how the relative outlier threshold (page 9) was chosen and the impact that different choices of this threshold would have on the result.
- Figure 7: I think it would be good to include the results of the first two models in this plot.

Minor comments:

- Second paragraph of the introduction: Although *outbreaker2* is acknowledged throughout the manuscript, I think it would be good to mention that *outbreaker2* is also highly customisable in the introduction. For example, I think (although I am not sure) that it should be possible to customise the contact function in *outbreaker2* to incorporate spatial information similar to *o2geosocial*.
- Fourth paragraph of discussion (page 20): I appreciate the authors' efforts to outline the limitation of using transmission trees/clusters to assess outbreak risks. This is a very minor point that the authors should feel free to ignore but I think this paragraph could be improved by suggesting that future models and implementations could incorporate additional data sources to address some of these issues.

Are the conclusions about the tool and its performance adequately supported by the findings presented in the article?

Partly

Is the rationale for developing the new software tool clearly explained?

Yes

Is the description of the software tool technically sound?

Yes

Are sufficient details of the code, methods and analysis (if applicable) provided to allow replication of the software development and its use by others?

Yes

Is sufficient information provided to allow interpretation of the expected output datasets and any results generated using the tool?

Yes

Reviewer Expertise:

Phylodynamics, pathogen genomics, applied statistics

I confirm that I have read this submission and believe that I have an appropriate level of expertise to confirm that it is of an acceptable scientific standard, however I have significant reservations, as outlined above.

# Peer review: Reviewer response for version 1

**DOI:** 10.5256/f1000research.31053.r83247

**Published:** 2021-05-14

**Authors:** Eben Kenah

**Affiliations:** 1 Biostatistics Division, College of Public Health, The Ohio State University, Columbus, OH, USA

While I appreciate the quality of the programming in o2geospatial and the detailed demonstration of its use, I think the interpretation of its results in infectious disease epidemiology is far from clear. The use of cluster size distributions to investigate the transmission of the disease is difficult because the apparent clusters are highly sensitive to the detection of cases, which is a particular problem if there are many subclinical cases as in influenza or COVID-19. The use of transmission tree reconstruction to understand risk factors for transmission is equally difficult because we are often lacking denominators (e.g., how many opportunities were there to transmit) and because a tree always has a mean outdegree just below one, which means that the censoring of secondary infections caused by infectious individuals in the later part of the observation period needs to be accounted for (e.g., see Fraser, PLOS One, 2007).

 The use case centers around a simulated data set whose generation is not clearly described, and it is often difficult to compare the model results to the "truth" contained in the simulation. The analyses of the simulated data use the same incubation period and serial interval distributions, so the analyses do not give us any sense of how the results would be affected by the misspecification of these distributions. This is mentioned briefly in the Discussion (to the authors' credit), but it is a fundamental shortcoming of the use case because these distributions are never known in practice.

**Comments:**

1. (page 4, paragraph second from bottom) "sampled form" should be "sampled from".
2. (page 5, conditional report ratio) It would be more accurate to say that the number of missing generations follows a geometric distribution with mean (1 - \rho) / \rho, not an exponential distribution.
3. (page 5, conditional report ratio) It is confusing to denote the conditional report ratio but then to refer to its prior distribution as prior_pi in the package. It would also be good to point out to readers that the prior distribution on the conditional report ratio is informative: It has a mean of 0.91 and a standard deviation of 0.08.
4. (page 5, time component) The definition of f_dens given in the text does not seem to match its definition in outbreaker_data.R, where it is defined as the "colonization time, i.e. the time interval during which the pathogen can be sampled from the patient". The incubation period is usually defined as the time between infection and the onset of symptoms, so it would be helpful to specify whether T_i is meant to be the time of the report (as in the text) or the reported symptom onset date (as implied by calling T_i - t_i an incubation period).
5. (page 5, time component) There are several problems that stem from the use of independent and identically distributed generation intervals as in the likelihood of Wallinga and Teunis (2004). First, generation intervals do not typically have the same distribution throughout an epidemic. They contract due to depletion of susceptibles and competing risks of infection from multiple sources. Thus, these likelihoods are only strictly correct in the very early stages of an epidemic (and only if there is negligible clustering of contacts). Second, it precludes an analysis of the effects of covariates on the risk of transmission between two individuals. Many critical questions in the response to an epidemic involve mechanisms of transmission and determinants of susceptibility and infectiousness. The proposed methods can address these questions only indirectly and on a coarse scale.
6. (page 6, description of simulated data) The simulations used to generate the data need to be described in more detail, including how the clusters were defined. The simulations themselves appear to be branching processes, which have several important differences from true stochastic epidemic models that should be mentioned.
7. (page 7, R code above Figure 2) The use of a normal distribution for a time-to-event distribution is surprising but not truly problematic. It might be good to call it something like a "normal distribution truncated at zero".
8. (page 8, top box) The R code in this box is inconsistent in the use of spaces when setting argument values. I believe the standard is to put spaces around the equals signs, which is followed through most of the paper.
9. (page 15, Figure 5) In Figure 4, the results from model 2 are the truth. In this figure, there is no obvious way of comparing the results of the models to any truth. The models produce very similar results, but do the estimated numbers of secondary cases correlate with an underlying true parameter such as a reproduction number?
10. (page 19 and earlier) "were ran" should be "were run".

Are the conclusions about the tool and its performance adequately supported by the findings presented in the article?

Partly

Is the rationale for developing the new software tool clearly explained?

Partly

Is the description of the software tool technically sound?

Yes

Are sufficient details of the code, methods and analysis (if applicable) provided to allow replication of the software development and its use by others?

Yes

Is sufficient information provided to allow interpretation of the expected output datasets and any results generated using the tool?

Partly

Reviewer Expertise:

Statistical methods for infectious disease epidemiology, mathematical models of epidemics, epidemiologic methods, causal inference, survival analysis

I confirm that I have read this submission and believe that I have an appropriate level of expertise to confirm that it is of an acceptable scientific standard, however I have significant reservations, as outlined above.

# Response

**Published:** 2021-05-27

**Authors:** Alexis Robert

**Affiliations:** London School of Hygiene & Tropical Medicine, UK

Thank you for your thoughtful comments on this paper. Please find our responses below

1. We agree that the limitations associated with the use of transmission trees should have been described in more detail. We have added a paragraph in the Discussion to list the main limitations associated with the use of cluster size distribution and transmission trees, and explain their value in infectious disease epidemiology.
  “The use of transmission trees and transmission clusters to assess current or future risk of outbreaks comes with various limitations. First, it relies upon the assumption that previous transmission patterns are representative of future outbreaks and requires previous incidence in the region of interest to be informative. Second, it requires past observed transmission events, and does not account for the number of opportunities of transmission per case. Where only sporadic isolated cases have been reported in the country, it is not possible to draw relevant conclusions on communities potentially most vulnerable to transmission. Third, partial detection of cases may bias the cluster size distribution, and under-estimate the number of secondary transmissions. Patterns of transmission, and characteristics associated with high-transmission events may still be observable but could introduce a bias if reporting is itself is affected by the same factors as is transmission. Finally, the use of transmission trees for real time modelling can be challenging, given the right-censoring of transmissions caused by recent infectious individuals ( *Fraser, 2007*).”
  Despite these limitations we believe our package is an useful contribution to the field: As we outlined in the Introduction, transmission trees have repeatedly been used in the recent years to investigate various aspects of the transmission dynamics, such as superspreading events, determinants of transmission, or the impact of various policies, including for pathogens where part of the cases was not detected ( *Wang et al Nature 2020, Wang & Teunis Frontiers in Medicine, 2020, James et al, PLOS One 2021*). The method we developed aims to improve the inference of transmission links between cases, so that information on outbreak dynamics can be deduced from transmission trees even in settings where they are not reconstructed through thorough contact tracing investigations.
2. We agree that it is important to highlight the impact of potential misspecifications of the input distributions. The first section of the Extended Data document describes the impact of changing the mean or the standard deviation of both distributions on the accuracy of the reconstructed transmission trees. This is now also described in the Discussion;
  “We re-ran the inference procedure using different distributions (changing the mean or the standard deviation), the results can be seen in the Extended Data. When the distributions were set with lower standard deviations, several links were not observed in the inferred transmission trees anymore. Indeed, these connections had been made impossible since the range of likely values was narrower. In all other examples, the simulated and inferred clusters size distribution remained very close, we only observed a slight drop in the proportion of iterations that contain the right transmission links. Since the likelihood of connection is computed from several components, the discrepancies between the distributions used in the simulations and the model fits did not substantially changed the inferred trees.”
3. This typo has been fixed, thank you for spotting it. 
4. We changed the distribution to a geometric distribution.
5. We changed the definition of the conditional report ratio in the main text to Π , in order to match the commands defined in *o2geosocial*. We added the following to clarify that the default prior distribution was informative and could be edited before running the model:
  “By default, the prior distribution is parametrised as Beta(10,1), which is an informative prior of mean 0.9 and standard deviation 0.08.The two parameters of the beta prior distribution can be changed using the variable prior_pi in create_config().””
6. Thank you for noticing this. Indeed, f_dens represents the incubation period, i.e. the time between the reported symptom date and the infection date. Therefore, T_i is the reported symptom onset date. We clarified this in the Main Text, and changed the description of the variable in outbreaker_data.R. This will be updated on CRAN at the next submission.
7. We agree that using independent and identically distributed generation intervals is a simplification, and does not represent the complexity observed throughout an epidemic. However, the temporal component of the likelihood function could be customised to generate different distributions of the generation intervals at different points of the outbreak. This would require implementing a new temporal likelihood function, which would use different values of w_dens depending on the time and regions , and adding it to the model using custom_likelihood(timing_infecttions = new_ll_timing()). We agree that in the current version of o2geosocial, the distributions used at different points would have to be defined prior to running the model, and could not be estimated as part of the fitting procedure. This will be an avenue for future development of the package, in order to seamlessly integrate new parameters to the models. We summarised these two points at the end of the “Customise the likelihood...” section:
  “In this use case, we only explored customising the spatial component. However, the other components of the likelihoods can also be edited, using the functions custom_priors(), custom_likelihoods(), or custom_moves(). For instance, to account for changes in the distribution of the generation time throughout an outbreak (Svensson 2007), one would have to change the element timing_infections of custom_likelihoods(). However, the distribution would need to be set prior to running the models.”
  In the Discussion we added
  “The default implementation of the method assumes that generation intervals are independent and identically distributed throughout an outbreak, whereas in reality, depletion of susceptibles and competing risk of infection through clustering of contacts would be expected to affect the generation interval. The method can be customised to integrate time varying generation intervals set prior to running the models. However, estimating the distribution of the generation interval during the inference procedure is more challenging to implement in the current framework, which may introduce a bias in our results.”
8. We added the following paragraph to to the Use Case section to describe how the simulated data were generated:
  “In order to assess whether the method was able to reconstruct the transmission links between cases, we needed to simulate the transmission trees. Population-level compartmental models cannot be used to generate who-infected-whom. Therefore, we generated the dataset at an individual level, by simulating different transmission trees in the area of interest. The transmission trees were generated using the following process:
  1.   We created an imported case, with random onset date, region of origin, and age group.
  2.   We drew the number of secondary cases stemming from this case, using a random reproduction number.
  3.   If the number of secondary cases was greater than 0, the characteristics of the new cases were drawn using the distributions of the generation time, incubation periods, the spatial kernel, and the proportion of contacts between age groups.
  4.   We repeated steps 2 and 3, for each new case, until no more secondary cases were drawn (i.e. the random reproduction number in step 2 was 0 for all new case).
  5.   We repeated steps 1 to 4, until we reached a maximum number of cases, or maximum number of trees, defined before the simulation.
  Numerous factors influencing the transmission dynamics are not included in this simulation framework. However, we do not aim to generate transmission trees which describe the spread of a given pathogen (here measles) in a community with complete accuracy. The main aim of this simulated dataset is to highlight the inference capabilities of the reconstruction method, and to explore causes for discrepancies between the simulations and the model fits, in an ideal setting where all parameters are known and are accounted for in the model.”
9. We changed how we refer to this distribution, we now use “normal distribution truncated at zero”. Thank you for this suggestion.
10. We fixed the use of spaces in this box.
11. Thank you for this suggestion. We have added a third panel to Figure 6, describing the average number of secondary cases per region in the simulations. Therefore, the results produced by both models can be compared to the data. We also added a comparison between the regional average number of secondary cases in the simulations and the 95% Credible Intervals of the models in the Extended data. We added the following sentences to describe Figure 6:
  “Similarly, we observe minor differences between the maps generated by the models and the simulated data. Most of the regions that repeatedly caused further transmissions in the simulations are identified by the models. In the Extended Data, we compared the regional number of secondary transmissions in the simulated data to the 95% Credible Intervals of both models, and found that the models were able to capture the input values in each region.”
12. These typos have been modified, thank you for spotting them.

# Peer review: Reviewer response for version 1

**DOI:** 10.5256/f1000research.31053.r82935

**Published:** 2021-04-21

**Authors:** Paige B. Miller

**Affiliations:** 1 Centers for Disease Control and Prevention, Atlanta, GA, USA

The authors present the R package, o2geosocial, for reconstructing transmission trees of infectious diseases that have slow mutation rates or incomplete genetic sequencing data. The model description is technically strong and similar to other tree reconstruction papers. I was able to easily verify and implement examples described in the paper using o2geosocial. The limitations of the package are adequately discussed throughout. Overall, I think package and paper are good additions to transmission tree reconstruction tools and literature. 

 I was wondering how flexible the package would be for including other types of data that researchers may want to include in the likelihood function, e.g., social groups, workplaces, shifts, etc. A brief discussion of these modifications in the "Customize the likelihood section" might help readers determine if this package would be helpful for them.

Are the conclusions about the tool and its performance adequately supported by the findings presented in the article?

Yes

Is the rationale for developing the new software tool clearly explained?

Yes

Is the description of the software tool technically sound?

Yes

Are sufficient details of the code, methods and analysis (if applicable) provided to allow replication of the software development and its use by others?

Yes

Is sufficient information provided to allow interpretation of the expected output datasets and any results generated using the tool?

Yes

Reviewer Expertise:

Infectious disease modeling, transmission trees, social contact networks, tuberculosis

I confirm that I have read this submission and believe that I have an appropriate level of expertise to confirm that it is of an acceptable scientific standard.

# Response

**Published:** 2021-05-27

**Authors:** Alexis Robert

**Affiliations:** London School of Hygiene & Tropical Medicine, UK

Thank you very much for these positive comments.

 Indeed, we agree that making the package more flexible to integrate other data routinely-collected in certain settings is an important area of improvement. This is one of the things we want to facilitate in future versions of *o2geosocial*. We added the following sentences to the Conclusion to discuss this aspect:

 “Future developments in the package will focus on facilitating the integration of new variables in the likelihood of connection, such as cases’ workplace or school. Currently, such variables would have to be integrated within one of the components of the likelihood. We aim to simplify the addition of new parameters and components in the inference framework.”
